# A shortest path-based approach for copy number variation detection from next-generation sequencing data

**DOI:** 10.3389/fgene.2022.1084974

**Published:** 2023-01-17

**Authors:** Guojun Liu, Hongzhi Yang, Xiguo Yuan

**Affiliations:** ^1^ School of Statistics, Xi’an University of Finance and Economics, Xi’an, China; ^2^ Medical Imaging Center, Xidian Group Hospital, Xi’an, China; ^3^ Hangzhou Institute of Technology, Xidian University, Hangzhou, China

**Keywords:** copy number variation, next-generation sequencing data, k nearest neighbors, shortest path, read depth

## Abstract

Copy number variation (CNV) is one of the main structural variations in the human genome and accounts for a considerable proportion of variations. As CNVs can directly or indirectly cause cancer, mental illness, and genetic disease in humans, their effective detection in humans is of great interest in the fields of oncogene discovery, clinical decision-making, bioinformatics, and drug discovery. The advent of next-generation sequencing data makes CNV detection possible, and a large number of CNV detection tools are based on next-generation sequencing data. Due to the complexity (e.g., bias, noise, alignment errors) of next-generation sequencing data and CNV structures, the accuracy of existing methods in detecting CNVs remains low. In this work, we design a new CNV detection approach, called shortest path-based Copy number variation (SPCNV), to improve the detection accuracy of CNVs. SPCNV calculates the k nearest neighbors of each read depth and defines the shortest path, shortest path relation, and shortest path cost sets based on which further calculates the mean shortest path cost of each read depth and its k nearest neighbors. We utilize the ratio between the mean shortest path cost for each read depth and the mean of the mean shortest path cost of its k nearest neighbors to construct a relative shortest path score formula that is able to determine a score for each read depth. Based on the score profile, a boxplot is then applied to predict CNVs. The performance of the proposed method is verified by simulation data experiments and compared against several popular methods of the same type. Experimental results show that the proposed method achieves the best balance between recall and precision in each set of simulated samples. To further verify the performance of the proposed method in real application scenarios, we then select real sample data from the 1,000 Genomes Project to conduct experiments. The proposed method achieves the best F1-scores in almost all samples. Therefore, the proposed method can be used as a more reliable tool for the routine detection of CNVs.

## 1 Introduction

As one type of structural variation, copy number variation (CNV) plays an important role in the formation and development of human cancers and diseases (Mccarroll and Altshuler, 2007; Stefansson et al., 2008; Beroukhim et al., 2010; Yuan et al., 2021a). Generally, CNV is defined as a deletion or amplification of a genomic sequence that is no less than 1,000 to several megabase pairs in length compared to a reference genome (Freeman et al., 2006; Zhao et al., 2013; Yuan et al., 2021b). The deletion and amplification of a copy number can lead to the reorganization of the genome structure and the change of base content, which further affects the level of human gene expression (Sebat et al., 2004; Sharp et al., 2005). Studies have shown that the occurrence of some common human diseases is closely related to CNV, such as ovarian cancer (Adam and David, 2009; Fridley et al., 2012), breast cancer (Tchatchou and Burwinkel, 2008; Kumaran et al., 2017), autism (Sebat et al., 2007; Pinto et al., 2010), schizophrenia (Stefansson et al., 2008; Stone et al., 2008), etc. In this context, next-generation sequencing (NGS) technology has developed rapidly and is able to provide rich data resources for the accurate detection of CNVs in the human genome, higher resolution, and more flexible detection methods (Meyerson et al., 2010). However, due to various factors, such as bias, noise, and the uneven distribution of NGS data, the existing detection methods are still not accurate for CNV detection.

A large number of CNV detection methods have been developed around NGS data, and the vast majority of them are based on the read depth (RD) method. The basic principle of RD-based CNV detection methods is that the number of reads aligned at each position in the reference genome is proportional to the copy number at that position (Yoon et al., 2009). Compared with the normal region, the number of reads in the copy number amplification region is higher, and the number of reads in the copy number deletion region is lower (Teo et al., 2012). The RD method can use single-end sequencing reads or paired-end sequencing reads to detect CNVs. In principle, it can detect the amplified and deleted regions of CNVs of any length. In practical applications, this method is more sensitive for detecting long CNVs. Therefore, it is more suitable for detecting copy number amplified regions but cannot accurately detect variant boundaries, resulting in detection results that contain a large number of false-positive positions.

The basic process of RD-based methods for detecting CNVs includes: 1) Reading the alignment and extracting RDs; 2) Preprocessing the RDs; 3) Building the detection model (statistical model, machine learning algorithm, etc.); 4) Selecting a reasonable threshold strategy and predicting CNVs. Based on the above workflow, some well-known RD-based CNV methods have been proposed, mainly including FREEC (Boeva et al., 2012), CNV-LOF (Yuan et al., 2021a), CNVnator (Abyzov et al., 2011), BIC-seq2 (Xi et al., 2016), SeqCNV (Chen et al., 2017), CNV_IFTV (Yuan et al., 2021b), and iCopyDAV (Dharanipragada et al., 2018). FREEC obtains the normalized read count profile by using GC-content or mappability profiles and employs a lasso-based algorithm to produce a smooth copy number profile that predicts genotype status for each genomic segment. FREEC is more sensitive to copy number gain regions than loss regions, and the detection results have a large number of false-positive positions, resulting in lower precision. CNV-LOF performs a segmentation procedure on the RD profiles to obtain consecutive and non-overlapping RD segment profiles. On this basis, a cyclic binary segmentation (CBS) algorithm (Venkatraman and Olshen, 2007) is performed on each segment to divide each one into a set of segments. CNV-LOF utilizes the idea of a local outlier factor to assign an outlier score for each RD segment. Based on the anomaly score profile, it predicts CNVs using a boxplot procedure. It is not sensitive to the detection of loss regions, and its performance is not well balanced between recall and precision. CNVnator calibrates the GC content to normalize the RD profile and uses a mean-shift approach to segment the RD profile to predict CNVs. CNVnator is able to detect a large number of long CNVs, the vast majority of which are false-positive events. Therefore, it achieves low precision, especially in the detection of low-purity samples. BIC-seq2 normalizes the RD profile at the nucleotide level and uses the bayesian information criterion to predict CNVs. While its performance is balanced between recall and precision, it has low precision in detecting high-purity samples. SeqCNV extracts the RD signal from paired samples, establishes a maximum penalized likelihood estimation model, and selects a threshold interval to predict CNVs. It is sensitive to short CNV detection and is not suitable for the detection of low-purity samples. CNV_IFTV utilizes the isolation forest algorithm to calculate an anomaly score for each RD, smooths the anomaly score profile using a total variation model, and uses the anomaly score to fit a gamma distribution to predict CNVs. The difference between the established statistical model and the actual distribution of RDs affects the accuracy of the CNV_IFTV detection. iCopyDAV automatically estimates bin size, calibrates GC-content and mappability bias using the median method and mappability score file, and performs segmentation using the CBS algorithm to predict CNVs. It is suitable for testing high purity and medium coverage samples. In general application scenarios, the above methods can effectively detect a large number of CNVs. However, their performance is uneven in the detection of samples of different purity.

With consideration of the above issues, we propose a new approach in this work to accurately detect CNVs using NGS data from the whole genome. The method is called shortest path-based CNV (SPCNV). The SPCNV calculates the k nearest neighbors of each RD and defines the shortest path, the shortest path relation, and the shortest path cost sets. Based on these three types of shortest path sets, we calculate the mean shortest path cost of each RD and its k nearest neighbors. A relative shortest path score equation is then built using the ratio between the mean shortest path cost of each RD and the mean of mean shortest path cost of its k nearest neighbors, which can calculate a score for each RD (Tang et al., 2002). Based on the score profile, a boxplot program is used to predict CNVs(Zijlstra et al., 2007). The main contributions of the proposed method are as follows: 1) According to the basic principle of the RD method, the copy number gain and loss correspond to larger and smaller RDs compared to normal RDs, respectively. The two types of RDs have fewer ratios among all RDs. Therefore, we treat the two types of RDs as outliers and successfully transform a traditional outlier detection method into a CNV detection method. 2) By extracting two features, the RD ratio and the difference between adjacent RD ratios, we can observe the difference between RDs from a global and local perspective, which is conducive to detecting isolated variants and local small cluster variants. 3) The proposed method uses the difference between the shortest path of each RD and the average shortest path of its k nearest neighbors to identify CNVs, which is beneficial for identifying a local cluster of insignificant variations. As the traditional machine learning method only relies on the distance between each RD to distinguish the difference between them, it cannot detect a small local cluster variation because their differences are very small.

The remainder of this work is organized as follows. Section 2 includes the workflow of SPCNV, data preprocessing, construction of relative shortest path score formula, and the forecasting of CNVs. Section 3 presents the simulation data and real data experiments and analyzes and discusses the experimental results. Section 4 addresses the shortcomings of the work and presents future work ideas.

## 2 Method and materials

### 2.1 Overview of SPCNV

**FIGURE 1:**
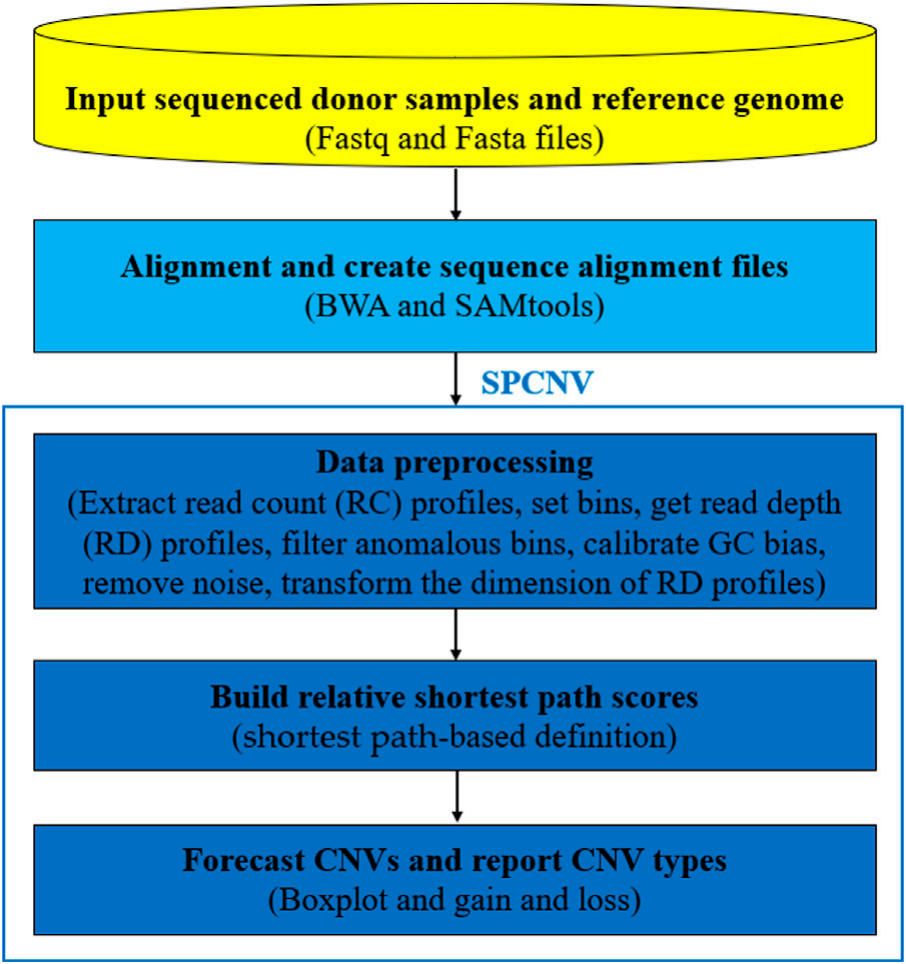
The workflow of SPCNV.

SPCNV is an RD-based CNV detection method that is suitable for the detection of a single sample. Figure 1 shows the workflow of SPCNV in detail, which consists of the following main five steps: 1) The sequenced donor samples (Fastq) and reference genome (Fasta) are prepared for input; 2) The reads are aligned to the reference genome using BWA (Li and Durbin, 2010) to generate sequence alignment files (SAM), which are converted to BAM format using SAMtools (Li et al., 2009); 3) The data is preprocessed. This step mainly includes read count (RC) profile extraction with SAMtools, bin definition (Yuan et al., 2018), anomalous bin removal, obtaining the read depth (RD) profiles, GC bias calibration, noise removal, and the dimension transformation of the RD profiles; 4) The relative shortest path score is built and assigned for each RD; 5) Based on the score profile, a boxplot is utilized to predict CNVs. The SPCNV software is developed in R and Python languages. It can be downloaded from https://github.com/gj-123/SPCNV/releases and is easy to install and use after reading the user manual. In the following section, each step in the workflow of SPCNV is analyzed and discussed in detail.

### 2.2 Data preprocessing

The sequenced donor samples are aligned to the reference genome using the BWA tool, which generates sequence alignment files in the SAM format. The SAM files are further converted into binary sequence alignment files in BAM format using SAMtools. The read count (RC) profiles are then extracted with SAMtools from the BAM files. We define a sliding window procedure (bin) (Yuan et al., 2018), with which the RC profiles are divided continuously and are non-overlapping to generate the RD profiles. This process is described using Eq. 1.

$$RD=\left\{RD_{1},RD_{2},RD_{3},\cdot \cdot \cdot ,RD_{n}\right\},$$ (1)

where $RD_{n}$ represents the RD value of the n-th bin, which is equal to the mean RC in a bin. Since the reference genome contains a large number of “N" positions, reads aligning to these positions will result in RCs equal to 0 that will be mistaken for a loss at that position. Therefore, if a bin contains “N" positions, we remove the bin from the genome sequence (Yuan et al., 2021a). Due to the complexity of the human genome, the distribution of GC-content is uneven, which can lead to the misidentification of copy number deletions. The GC content of each bin is calibrated using the median method (Yoon et al., 2009). Factors such as alignment errors and biases can cause the resulting sequencing data to be noisy. RD signal noise will seriously affect the detection accuracy, which is a key step in the detection of CNV. Here, the total variation model (Condat, 2013; Duan et al., 2013) is used to smooth and segment the RD profile to generate an RD segment (RDS) profile, which is represented by Eq. 2.

$$RDS=\left\{RDS_{1},RDS_{2},RDS_{3},\cdot \cdot \cdot ,RDS_{n}\right\},$$ (2)

where $RDS_{n}$ represents the value of the n-th read depth segment, which is equal to the mean of all RDs contained in this segment. The RDS profile is converted to two-dimensional space to generate the $RDS^{\prime }$ profile (Liu et al., 2020), which is composed of the RDS ratio and differences between adjacent RDS ratios and is expressed by Eq. 3.

$$RDS^{\prime }=\left\{\left(RDSX_{i},RDSY_{i}\right)|i\in N,1\leq i\leq n\right\},$$ (3)

where $RDSX_{i}$ represents the value of the i-th RD ratio, $RDSY_{i}$ represents the difference between the i-th RD ratio and its adjacent RD ratios.

This transformation process can detect differences in RD from two perspectives. The first dimension can approximately reflect the copy number status corresponding to each RD from a global perspective. The second dimension can approximately reflect the difference between an RDS and its adjacent RDSs from a local perspective. By extracting two features of RD, the proposed method can more easily discover globally isolated and local small cluster variants. At the same time, this step also provides an effective data platform for constructing the relative shortest path score in the next section.

### 2.3 Establishment of relative shortest path score

Based on $RDS^{\prime }$ profile, we construct a relative shortest path score (RSPS) to evaluate the degree of anomaly of each RDS. Here, we regard each element in $RDS^{\prime }$ as an object represented by *o*. The RSPS fully reflects the closeness between an object and its surrounding objects and is highly suitable for application in CNV detection scenarios. The RSPS of an object depends on the ratio between the object’s shortest path and the mean of its k nearest neighbors’ shortest paths. Some related basic concepts and definitions must be introduced before giving the definition of RSPS, which mainly include the k-distance of an object, the k-distance neighborhood of an object (Breunig et al., 2000), the shortest path set, the shortest path relation set, and the shortest path cost set.

Definition 1 The *k*-distance of an object *o,* which is defined using Eq. 4.

$$k-dist\left(o\right)=dist\left(o,o^{\prime }\right),$$ (4)

where $dist\left(o,o^{\prime }\right)$ represents euclidean distance between object o and object $o^{\prime }\in RDS^{\prime }\backslash \left\{o\right\}$ , $o^{\prime }$ indicates that the k-th object closest to *o* is sorted in ascending order, $k-dist\left(o\right)$ represents the *k*-distance of an object *o*. Here, k is a positive integer.

Definition 2 The *k*-distance neighborhood of an object *o* is a collection of objects whose distance from *o* is less than or equal to $k-dist\left(o\right)$ , which is defined using Eq. 5.

$$N_{k-dist}\left(o\right)=\left\{a|a\in RDS^{\prime }\backslash \left\{o\right\},dist\left(o,a\right)\leq k-dist\left(o\right)\right\},$$ (5)

where $N_{k-dist}\left(o\right)$ represents the *k*-distance neighborhood of an object *o* and a collection of objects, and the distance between each object in the collection and o is not greater than $k-dist\left(o\right)$ .

The shortest path set (SPS) of an object *o* is composed of object *o* and the *k*-distance neighborhood of object *o*, which are connected to form a path with the shortest distance. The shortest path relation set (SPRS) of an object *o* is defined as the edge between two objects on the shortest path. The shortest path cost set (SPCS) of an object *o* is defined as the distance between two objects on the shortest path. Algorithm 1 describes the calculation process of SPS(*o*), SPRS(*o*), and SPCS(*o*) in detail.

**FIGURE 2:**
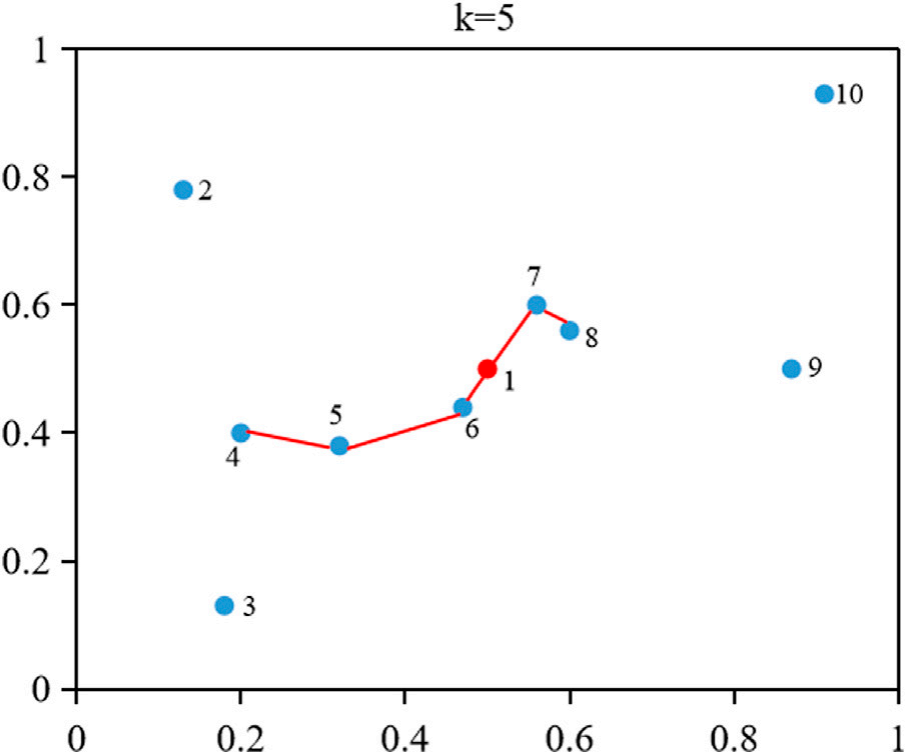
An example of calculating the SPS, SPRS, and SPCS of object 1.

The following example is used to clearly explain the SPS, SPRS, and SPCS calculation process of an object. As shown in Figure 2, there are a total of 10 objects {1, 2, 3, 4, 5, 6, 7, 8, 9, 10}, and their corresponding values are {(0.5, 0.5), (0.13, 0.78), (0.18, 0.13), (0.2, 0.4), (0.32, 0.38), (0.47, 0.44), (0.56, 0.6), (0.6, 0.56), (0.87, 0.5), and (0.91, 0.93)}. We calculate the SPS, SPRS, and SPCS of object one here, where the value of k is set to 5. Eq. 4 and 5 are employed to obtain the five nearest neighbors of object 1 (6, 7, 8, 5, 4). The distance from object one to object six is the smallest. According to Algorithm 1, SPS (1) is equal to {1, 6}, SPRS (1) is equal to {(1, 6)}, and SPC S(1) is equal to {0.07}. The distance between objects 7, 8, 5, 4, and objects 1, six is calculated to obtain a minimum distance, and the distance between objects one and seven is the smallest. Similarly, SPS (1) is equal to {1, 6, 7}, SPRS (1) is equal to {(1, 6), (1, 7)}, and SPCS (1) is equal to {0.07, 0.12}. The procedure ends when each neighbor of object one finds its closest object from SPS(1). Finally, SPS (1) is equal to {1, 6, 7, 8, 5, 4}, SPRS (1) is equal to {(1, 6), (1, 7), (7, 8), (6, 5), (5, 4)}, and SPCS (1) is equal to {0.07, 0.12, 0.06, 0.16, 0.12}. As shown in Figure 2, the red line segments form the final shortest path.

Algorithm 1 Calculate SPS, SPRS and SPCS of object *o*. 
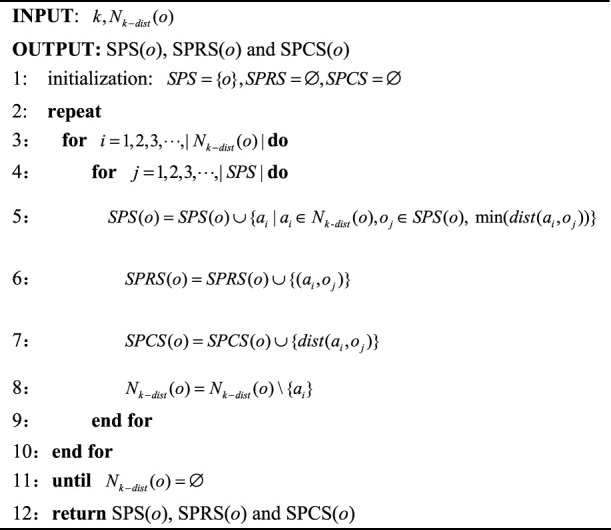

Based on the above definitions, the mean shortest path cost of an object *o* ( $SPC_{m}\left(o\right)$ ) is defined by Eq. 6.

$$SPC_{m}\left(o\right)=\frac{1}{k}\overset{k}{\underset{i=1}{\sum }}SPCS_{i}\left(o\right),$$ (6)

where $SPCS_{i}\left(o\right)$ represents the i-th element in $SPCS\left(o\right)$ .If the value of $SPC_{m}\left(o\right)$ is larger, the distance between *o* and its k nearest neighbors is sparser; if it is smaller, they are closer together.

After estimating the mean shortest path cost of all objects, we further construct the relative shortest path score (RSPS) to measure the degree of deviation between object *o* and its k nearest neighbors, which is defined by Eq. 7.

$$RSPS\left(o\right)=\frac{\left|N_{k-dist}\left(o\right)\right|\cdot SPC_{m}\left(o\right)}{\underset{a\in N_{k-dist}\left(o\right)}{\sum }SPC_{m}\left(a\right)},$$ (7)

where $SPC_{m}\left(o\right)$ represents the mean shortest path cost of *o*, $SPC_{m}\left(a\right)$ represents the mean shortest path cost of one of its nearest neighbors, $\left|N_{k-dist}\left(o\right)\right|$ represents the number of elements in $N_{k-dist}\left(o\right)$ , $RSPS\left(o\right)$ represents the ratio between $SPC_{m}\left(o\right)$ and the mean of mean shortest path cost of its k nearest neighbors. If the value of $RSPS\left(o\right)$ is larger, the distance between *o* and its k nearest neighbors is sparser; if it is smaller, they are closer together. This means that the higher the RSPS of an object, the more likely the object is a CNV.

### 2.4 Prediction of CNVs

Although we have evaluated the relative shortest path score for each object, it is not yet possible to distinguish abnormal objects from normal objects. This step is critical in CNV detection, and a reasonable threshold selection strategy will significantly improve the accuracy of the detection results. Traditional threshold selection strategies mainly include: 1) Fitting a statistical model using the score profile to evaluate the significance of each object and using hypothesis testing to predict CNVs; 2) Selecting an empirical value as a threshold to identify CNVs. The limitation of the first strategy is that due to the bias and noise of the sequencing data, the actual distribution of the data and the fitted model are quite different, resulting in inaccurate detection results. The limitation of the second strategy is that a fixed threshold can effectively identify abnormal objects in some scenarios. However, the performance of the method may drop significantly in certain application scenarios. Considering the above two scenarios, we use a boxplot to determine thresholds based on the score files. This method does not require an assumption that the score profile obeys a certain distribution in advance and is able to dynamically determine thresholds according to different score files. Here, we use Eq. 8 to estimate a threshold for judging anomalies.

$$\tau =RSPS_{Q_{3}}+\lambda \cdot \left(RSPS_{Q_{3}}-RSPS_{Q_{1}}\right),$$ (8)

where $RSPS_{Q_{3}}$ represents upper quartile of RSPS, $RSPS_{Q_{1}}$ represents lower quartile of RSPS, λ represents multiple of interquartile range of RSPS, τ represents the maximum value of the inner limit of RSPS, which is used as the threshold. If an object’s RSPS is greater than the threshold τ , it is considered to be a CNV. After predicting CNVs, we further differentiate the types of CNV (gain and loss). If the RD of an object is greater than or equal to the average RD of all normal objects, it is regarded as a gain; if it is less, it is regarded as a loss.

## 3 Results and discussion

Along with the establishment of SPCNV, the design of a reasonable experimental scheme is crucial for verifying the effectiveness of the proposed method. In this study, the experimental component was divided into simulation and real data experiments. In the simulation data experiments, the performance of the proposed method was compared with four well-known similar methods (CNV-LOF, FREEC, CNVnator, and BIC-seq2) from five perspectives: recall, precision, F1-score, the number of gain and loss detections, and sensitivity of different size CNV detection. In order to verify the performance of the proposed method in real data applications, the above four comparison methods were also selected for comparison with the proposed method. The experimental data was a set of real human sequencing samples from the 1000 Genomes Project. Some previous studies have tested these samples and saved the test results to the Database of Genomic Variants (DGV), which was used as ground truth to calculate recall, precision, and F1-score for each method.

### 3.1 Application of simulation data

IntSIM (Yuan et al., 2017) simulation software was adopted to generate the simulation data sets. Before using the software, the two key parameters of sample tumor purity (TP) and sequencing coverage (SC) were set from 0.2 to 0.8 and 5x, respectively. To ensure the reliability of the test results, 50 samples were generated under each set of configuration conditions and the average of which was used as the test result. There were six gains and eight losses embedded in each sample, whose lengths range from 10,000 to 50,000 bp.

**FIGURE 3:**
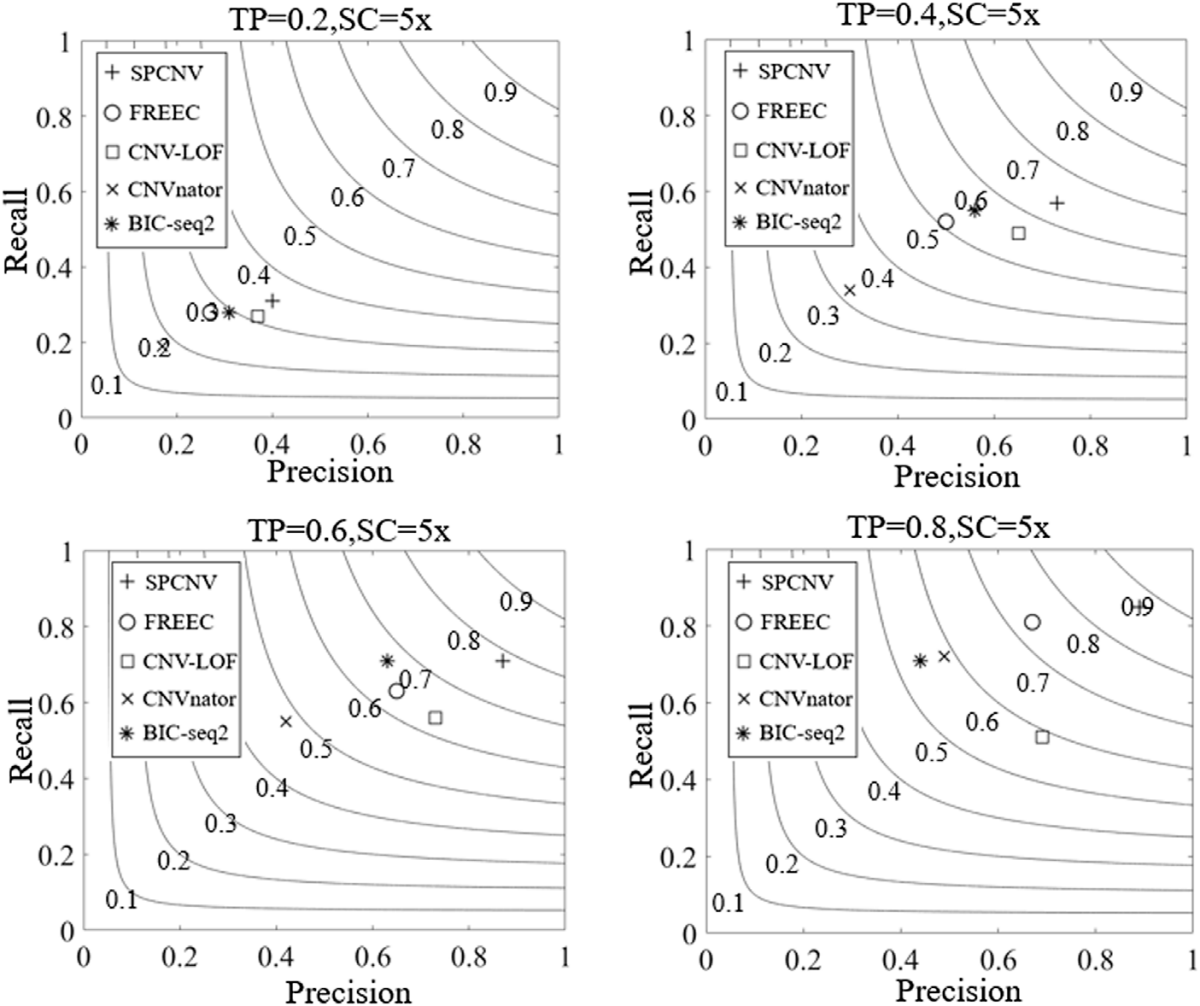
The performance of the five methods is compared in terms of recall, precision, and F1-score across four sets of simulation samples. Black curves indicate that the F1-score levels are harmonic means of recall and precision ranging from 0.1 to 0.9. The equations on the left and right sides of the comma represent the tumor purity (TP) and sequencing coverage (SC), respectively.

Based on the simulated datasets, the performance of SPCNV and four other alignment methods (CNV-LOF, FREEC, CNVnator, and BIC-seq2) were tested by calculating their recall, precision, and F1-scores. Recall is defined as the number of correctly detected CNV events divided by the total number of simulated CNV events, which can be calculated by the ground truth file (Magi et al., 2013). Precision is defined as the number of correctly detected CNV events divided by the total number of detected CNV events (Magi et al., 2013). The F1-score is defined as the harmonic mean of recall and precision. The experimental results of each method are depicted in Figure 3, where the three-performance metrics (recall, precision, and F1-score) of each method are compared in the four simulation sample sets. According to the overall trend, the performance of the majority of methods improves with increasing tumor purity. For example, the recall of CNVnator is close to 0.2 when the tumor purity is equal to 0.2, and its recall exceeds 0.7 when the tumor purity is equal to 0.8. Correspondingly, its F1-score increases from 0.18 to 0.58. Among the five methods, SPCNV achieves the best F1-score in each dataset. BIC-seq2 obtains the lowest F1-score at a purity equal to 0.8, but its F1-scores are better than other three methods (CNV-LOF, FREEC, and CNVnator) at a purity equal to 0.6. The above situations indicate that BIC-seq2 can provide base level resolution and detect a large number of CNVs, but its precision is very low when detecting high-purity samples. BIC-seq2 is not suitable for the detection of high-purity samples. The F1-score of FREEC is better than other three methods (CNV-LOF, BIC-seq2, and CNVnator) when the tumor purity is equal to 0.8. When detecting low and medium purity samples, its performance is relatively balanced between recall and precision. When FREEC detects high-purity samples, its F1-score is superior to the other three comparison methods, but its recall is significantly higher than precision, which indicates that its performance in detecting high-purity samples is uneven. The F1-scores of CNV-LOF are better than other three methods (BIC-seq2, FREEC, and CNVnator) when the tumor purity is equal to 0.2 and 0.4, which indicates that it is suitable for detecting low and medium purity samples. Its advantage is to obtain high precision when detecting low and medium purity samples. In contrast, its recall rate is low. CNVnator obtains the lowest F1-scores at purities equal to 0.2, 0.4, and 0.6, because the precision of CNVnator is the lowest in all sample sets. CNVnator detects a large number of long CNVs, most of which are false-positive positions. In terms of recall, SPCNV achieves the best recall, except when the tumor purity is equal to 0.6. In terms of precision, SPCNV obtains the best precision in each sample set. Overall, the SPCNV gets the best trade-off between recall and precision in each simulation sample set.

**FIGURE 4:**
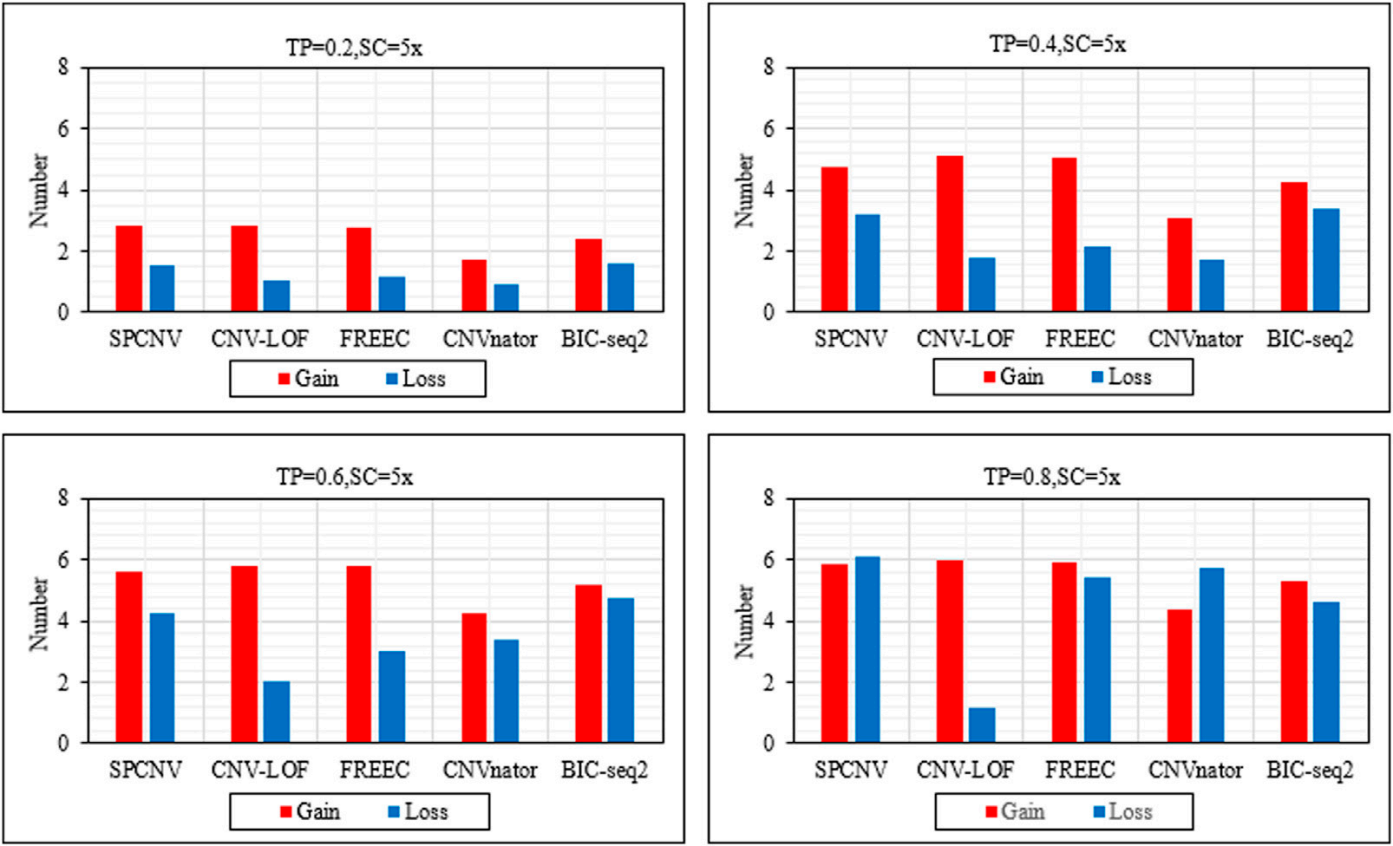
The performance of the five methods is compared in terms of the number of detected gains and losses across four sets of simulation samples. The equations on the left and right sides of the comma represent the tumor purity (TP) and sequencing coverage (SC), respectively.

Based on the above analysis and discussion, we further analyze the performance of each method in detecting gains and losses. Figure 4 details the performance of each method in detecting gains and losses in the four datasets. In general, the number of gains detected by each method is more than losses. The performance of SPCNV in detecting the gain and loss is relatively balanced compared to the other four methods. While CNV-LOF detects the most gains in each sample set, it obtains the least losses in the vast majority of cases. In most cases, FREEC detects far more gains than losses. CNVnator detects the least gains in each sample set and obtains fewer losses than most methods. BIC-seq2 detects the most losses in three sets of samples, indicating that it is suitable for detecting losses. In summary, SPCNV detects more gains and losses than most methods in each simulation sample set, which shows that its performance is robust in gain and loss detection.

**FIGURE 5:**
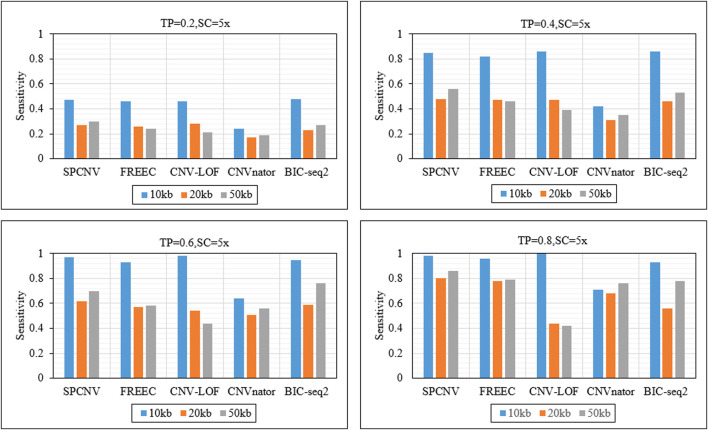
The sensitivity of five methods at the three CNV length levels under four sets of simulation configurations. The equations on the left and right sides of the comma represent the tumor purity (TP) and sequencing coverage (SC), respectively.

As a supplement to the above experiments, we further analyzed the sensitivity of each method to detect CNV of different lengths. Figure 5 details the sensitivity of five methods to detect three CNV lengths (10 kb, 20 kb and 50 kb) in four sets of samples. The sensitivity is defined as the ratio between the number of correctly detected CNVs and the number of simulated CNVs. The proposed method achieves the best sensitivity when the TP is equal to 0.2 and the CNV length is equal to 50 kb, and ranks second when CNV sizes are equal to 10kb and 20 kb. BIC-seq2 and CNV-LOF get the best sensitivity when the TP is equal to 0.2 and CNV sizes are equal to 10 kb and 20 kb. CNV-LOF achieves lower sensitivity at CNV sizes equal to 10 kb and 50 kb than other three comparison methods (SPCNV, FREEC and BIC-seq2). BIC-seq2 achieves lower sensitivity at CNV sizes equal to 20 kb and 50 kb than SPCNV. The proposed method gets the best sensitivity when the TP is equal to 0.4 and CNV sizes are equal to 20 kb and 50 kb, and ranks second when CNV size is equal to 10 kb. CNV-LOF and BIC-seq2 get the best sensitivity when CNV size is equal to 10 kb, and achieves lower sensitivity at CNV sizes equal to 20 kb and 50 kb than the proposed method. The proposed method gets the best sensitivity when the TP is equal to 0.6 and CNV size is equal to 20 kb, and ranks second when CNV sizes are equal to 10 kb and 50 kb. At the same time, CNV-LOF and BIC-seq2 get the best sensitivity when CNV size is equal to 10 kb and 50 kb, respectively. CNV-LOF achieves lower sensitivity at CNV sizes equal to 20 kb and 50 kb than SPCNV. BIC-seq2 achieves lower sensitivity at CNV sizes equal to 10 kb and 20 kb than SPCNV. The proposed method gets the best sensitivity when the TP is equal to 0.8 and CNV size are equal to 20 kb and 50 kb, and ranks second when CNV size is equal to 10 kb. CNV-LOF gets the best sensitivity when CNV size is equal to 10 kb, and achieves the lowest sensitivity at CNV sizes equal to 20 kb and 50 kb. In general, the proposed method performs better than the other four comparison methods in detecting CNVS of different sizes.

### 3.2 Application of real data

**FIGURE 6:**
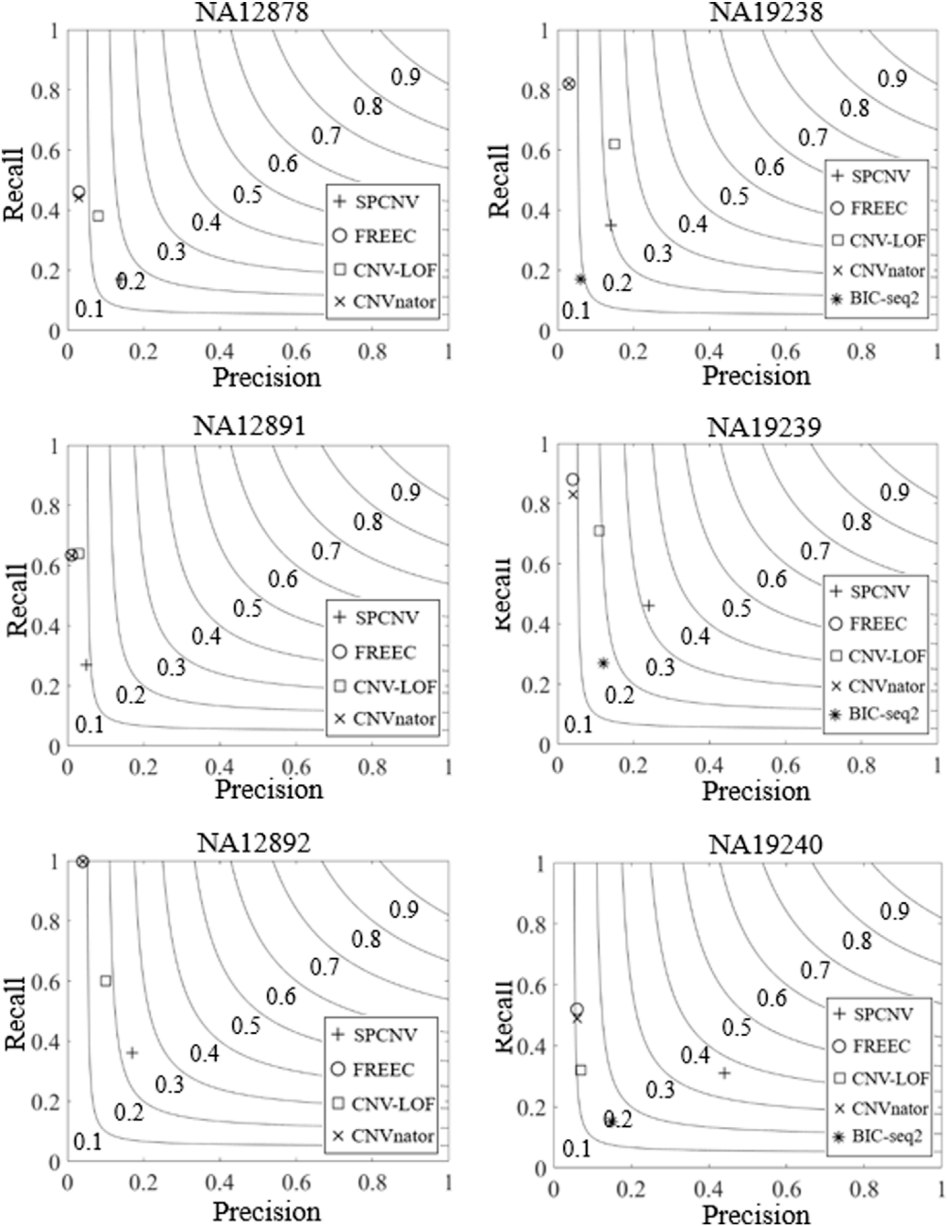
The performance of the five methods is compared in terms of recall, precision, and F1-score across six real data samples. Black curves indicate F1-score levels that are harmonic means of recall and precision ranging from 0.1 to 0.9.

In order to verify the performance of the proposed method in real application scenarios, we use six real data samples (NA12878, NA12891, NA12892, NA19238, NA19239, and NA19240) from the 1,000 Genomes Project, which can be downloaded for free from http://www.internationalgenome.org/. Some of the test results for these samples are recorded in the DGV, which is considered the ground truth for calculating the recall, precision, and F1-scores for each method. Similarly, we select the four methods (CNV-LOF, FREEC, CNVnator, and BIC-seq2) of the above experiments to compare with the proposed method. The experimental results are shown in Figure 6. SPCNV obtains the highest F1-scores among the five samples and ranks second in the NA19238 sample. CNV-LOF obtains the highest F1-score in the NA19238 sample and ranks second in F1-score among the other five samples. BIC-seq2 does not detect the correct CNVs in NA12878, NA12891, and NA12892, and its F1-scores rank third in NA19238, NA19239, and NA19240. The performance of FREEC and CNVnator is relatively close, and their F1-scores are ranked third and fourth in NA12878, NA12891, and NA12892 and in NA12878, NA12891, and NA12892, respectively. In terms of recall, FREEC achieves the best recall three times, CNVnator has the best recall twice, and CNV-LOF achieves the best recall once. In terms of precision, SPCNV obtains the best precision in five of the six samples, and CNV-LOF has the best precision once. Overall, SPCNV has obvious advantages over the other four methods in terms of precision and F1-score.

## 4 Discussion and conclusion

In this work, a new method called SPCNV was proposed to detect CNVs using NGS data. SPCNV was developed based on the RD method and could be used to detect a single genome-wide sample. The proposed SPCNV method effectively removed abnormal bins, calibrated GC-content bias, and denoised and transformed the dimension of the read depth. Based on the preprocessed RD profile, it then computed the k nearest neighbors for each object. On this basis, we constructed the shortest path set, the shortest path relation set, and the shortest path cost set for each object, by which the mean shortest path cost was defined. At the same time, we computed the mean shortest path cost for each object and used the ratio between the mean shortest path cost of each object and the mean of the mean shortest path cost of its k nearest neighbors to construct a relative shortest path score. Based on the relative shortest path scores for each object, CNVs were predicted using boxplots. Both simulation and real data experiments were then carried out to verify the performance of the proposed method. In the simulation data experiments, the performance of the proposed method was evaluated from five aspects (recall, precision, F1-score, the number of gain and loss, and sensitivity of detection of CNV with different sizes). The experimental results showed that the proposed method achieved the best balance between recall and precision, gain and loss, as well as CNV of different sizes, respectively. In real data applications, the proposed method achieved the best F1-scores in most samples, indicating that its performance was reliable and effective in real application scenarios.

Traditional CNV detection methods generally assume in advance that the RDs obey a statistical model, use the model to calculate a *p*-value for each read depth, and use hypothesis testing methods or select a fixed threshold to predict CNVs. Compared with the traditional methods, the proposed method has three different characteristics, which are summarized as follows. 1) Compared with traditional density-based methods, using objects to construct shortest paths can effectively identify a small cluster of local variants, which have little difference but are isolated relative to all objects. 2) We treat a CNV as an outlier and effectively transform the outlier detection method into a CNV detection method. 3) Extracting the RD ratio and difference of the RD ratio provides a global and local perspective to capture the difference of the copy number corresponding to each RD, which is more conducive to the detection of local single isolated CNVs and local small clusters of CNVs.

Although the performance of the proposed method meets the detection needs to a certain extent, there are still some shortcomings that require improvement. At this stage, the resolution of the proposed method must be further improved. In the next step, we will extract the read depth and split reads to further enhance its resolution (Ye et al., 2009; Jiang et al., 2012). The selection of the number of nearest neighbors is a key step that can affect the accuracy of the detection results. In this study, the selection of this parameter is based on reference to previous studies (Breunig et al., 2000; Jin et al., 2006). While the performance of the method is good in most application scenarios, it may not be suitable for some individual cases. In future work, the selection method of this parameter will be improved to realize automatic optimization. In addition, the functions of the proposed method need to be further expanded for application to more scenarios. For example, analyzing the biological significance of CNVs and mapping oncogenes, which can provide strong support for targeted drug development and clinical treatment.

## Data Availability

Publicly available datasets were analyzed in this study. This data can be found here: http://www.internationalgenome.org/.
