# Supplementary material for: A shortest path-based approach for copy number variation detection from next-generation sequencing data
Source: Front Genet. 2023 Jan 17;13:1084974. doi: 10.3389/fgene.2022.1084974 (PMC9887524; doi:10.3389/fgene.2022.1084974)

Supplementary Material

# Supplementary Tables

**Supplementary Table 1.** Performance analysis of the proposed methods under different sliding window sizes.

| Sliding window size | 1000 | 2000 | 3000 | 4000 | 5000 |
| --- | --- | --- | --- | --- | --- |
| Recall | 0.76 | 0.85 | 0.74 | 0.71 | 0.66 |
| Precision | 0.65 | 0.89 | 0.93 | 0.92 | 0.89 |
| F1-score | 0.7 | 0.87 | 0.82 | 0.8 | 0.76 |

# Obtain the screenshot of the software package


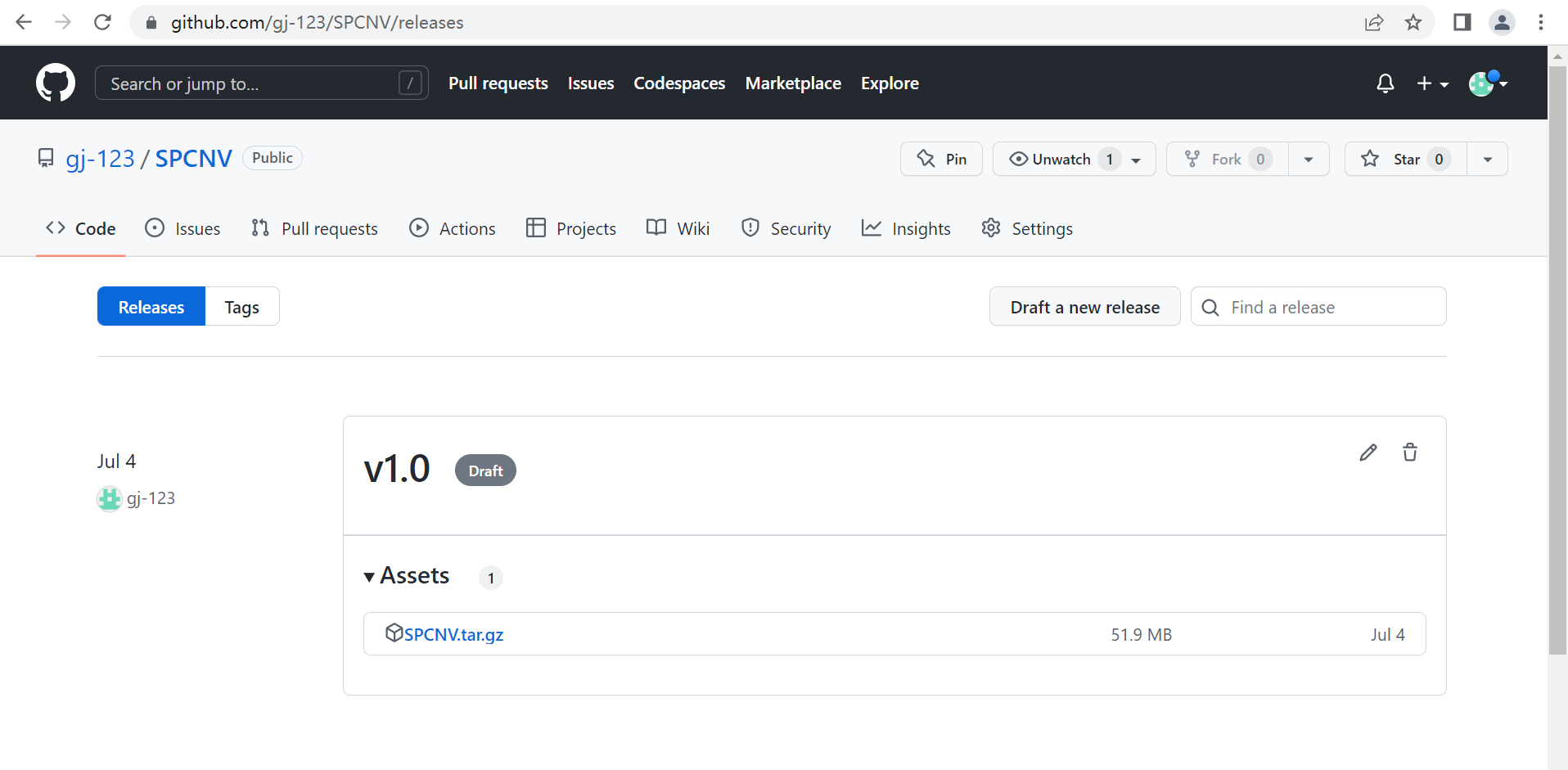

Supplement: Supplementary file 1 [file Table1.DOCX]
